# Effects of Including Gender Pronoun Questions in Surveys

**DOI:** 10.3389/fpsyg.2022.873442

**Published:** 2022-05-09

**Authors:** Adam Palanica, Luke Lopez, Amy Gomez, Yan Fossat

**Affiliations:** ^1^Klick Applied Sciences, Klick Health, Toronto, ON, Canada; ^2^People Practices, Klick Health, Toronto, ON, Canada; ^3^Diversity Strategy, Klick Health, Toronto, ON, Canada

**Keywords:** gender pronouns, gender minorities, sexual orientation, survey design, participant satisfaction, sex, gender, transgender

## Abstract

This research examines whether the mere presence of asking about gender pronouns (e.g., she/her, he/him, they/them, and ze/zir) in a survey enhances participants’ attitudes and satisfaction of answering the questions. A large sample (*N* = 1,511) of heterosexual, cisgender, and LGBTQIA+ participants across the United States (US) were surveyed an online “personality test” (as a deception), with the real purpose of examining whether asking a pronoun question enhanced their perceptions of the survey. Three demographic groups were included: (i) heterosexual–cisgender (*n* = 503), (ii) gay–cisgender (*n* = 509), and (iii) genderqueer (trans, non-conforming, other, *n* = 499). Half of each group were randomly given either a survey that included a gender pronoun question (test) or not (control), and then all rated their perceptions of the survey questions. For participants who identified as heterosexual or gay, no major differences were found between survey conditions. However, participants who identified as genderqueer experienced significant increases of satisfaction, comfort level, and perceived relevance of the questions when given a survey that asked their gender pronouns versus the survey that did not. These findings have implications for any surveys that ask about personal demographics, and suggest that any form of written communication should include clarity about gender pronouns.

## Introduction

Most surveys, such as healthcare forms, insurance applications, employee information sheets, financial documents, and scientific or market research questionnaires, typically ask participants about basic demographic questions, such as age, sex, and ethnicity to understand the sample population. However, rarely do these surveys ask about sexual orientation and gender identity (SOGI), which can tremendously enhance the value of data collection, as well as increase the satisfaction level and inclusiveness of the participants completing the survey. For all research and reporting purposes, it can be important to understand the unique characteristics of all heterosexual, cisgender individuals as well as lesbian, gay, bisexual, transgender, queer, intersex, asexual, and all sexual and gender minority (LGBTQIA+) members. Previous findings have demonstrated that the use of specific language influences the salience of social identity, which can further impact one’s own gender identity (Wigboldus et al., 2000; Moscatelli and Rubini, 2011; Beukeboom, 2014; Rubini and Menegatti, 2014; Tate et al., 2014).

Asking SOGI questions can be especially important within healthcare settings, since LGBTQIA+ members suffer from many healthcare disparities and receive poorer quality treatment compared to heterosexual and cisgender individuals. Research has shown that sexual and gender minorities have higher rates of drug, tobacco, and alcohol use, higher rates of high rates of mental and behavioral health issues, anxiety disorders, depression, and suicide attempts, and are less likely to receive preventive screenings and tests for cancers (National Academies of Sciences, Engineering, and Medicine, 2020; Radix, 2020; Statistics Canada, 2020).

LGBTQIA+ members are also more prone to challenging and hostile work environments with greater discrimination compared to their heterosexual, cisgender counterparts (Button, 2001; Badgett et al., 2007). This can elicit identity-safety concerns and fear that their identity will not be valued or accepted, along with discomfort and mistrust in that organization (Johnson et al., 2021). By contrast, workplaces that embrace organizational practices supporting LGBTQIA+ employees are associated with higher feelings of belonging and acceptance, greater job satisfaction, and organizational commitment (Button, 2001; Griffith and Hebl, 2002; Köllen, 2015).

Additionally, with the advance of recent social movements, such as “Me Too” (#MeToo), and “Black Lives Matter” (BLM), there is a heightened awareness of mistreatment toward minority demographic groups in society, which should be rectified to foster a better culture of Diversity, Equity, and Inclusion (DEI). Creating an inclusive culture toward all members of society can help mitigate discrimination within government, healthcare, workplaces, and education.

Previous research has shown that including SOGI questions in healthcare intake forms is acceptable, relevant, and important to patients and providers, especially since sexual and gender minorities experience significant health disparities and require care and services tailored to their unique needs (Cahill et al., 2014; Thompson, 2016; Dichter et al., 2018; Haider et al., 2018; Rullo et al., 2018; Pinto et al., 2019; Puckett et al., 2020; Lau et al., 2021). Importantly, these findings suggest that most individuals are not offended by SOGI questions. Although research has strongly recommended the inclusion of asking detailed questions about gender identity, many surveys still fail to do so (Westbrook and Saperstein, 2015; Grasso et al., 2019; Guss et al., 2020; Suen et al., 2020). Demographic questions usually involve a simple binary choice of “female” or “male”, but this can lead to inaccurate responses and discomfort for participants who are misaligned with their biological sex at birth and social gender identity, or are confused about their own personal beliefs and sexual orientation (Morgenroth and Ryan, 2020). It is also rare that any survey materials include questions about gender pronouns, such as “she/her,” “he/him,” “they/them,” or “ze/zir.”

Little systematic research has examined whether including pronouns or not during written communication can induce feelings of comfort and respect toward either LGBTQIA+ members or heterosexual and cisgender individuals. One study gave LGBTQ+ participants fictitious employee biographies to examine their impressions of workplace organizations (Johnson et al., 2021). The biographies were manipulated to include either gender pronouns or not. The experiment showed that employee biographies which explicitly identified the employee’s gender pronouns resulted in more positive attitudes toward that workplace, and greater organizational attraction, commitment, and trust, relative to the biographies with pronouns absent. Another experimental study assessed participant attitudes toward gender pronouns and other SOGI questions when given a healthcare survey (Rullo et al., 2018). In that study, 491 patients from a large academic medical center were randomly assigned to complete either intake forms with SOGI questions (experimental) or intake forms without SOGI questions (control). Experimental questions included sex assigned at birth, current gender identity, sexual orientation, and gender pronoun. The findings showed no significant differences in patient attitudes between experimental and control groups. However, only 0.8% of that patient sample identified as lesbian, gay, and bisexual, and no participants identified as transgender. Thus, that particular study was limited in its generalizability beyond the heterosexual and cisgender population, and it is unclear whether a survey that asks LGBTQIA+ individuals about their pronouns would elicit more positive and empathetic perceptions of taking that survey overall.

An estimated 4.5% of the United States (US) population identifies as lesbian, gay, bisexual, or transgender (Conron and Goldberg, 2020). The current study helps extend previous research of attitudes toward pronoun survey questions by examining a wide range of English-speaking LGBTQIA+ and heterosexual, cisgender individuals across the US. This research implemented a test/control experimental study, in which participants were given a survey under the guise of a “personality test for research purposes” to mitigate response bias. The survey comprised a demographics question page with two different versions: one that included a gender pronoun question (test), and one that did not (control). The true purpose of the study was to examine how satisfied and comfortable participants felt after they completed the survey, and whether the mere presence of asking a single pronoun question (e.g., she/her, he/him, they/them, and ze/zir) enhanced their attitudes of taking the survey.

## Materials and Methods

### Participants and Recruitment

A cross-sectional sample of 1,511 US participants completed the survey (Table 1). Participants were gathered from a large national sample using the online survey platform Prolific,^1^ which is comprised of a wide distribution of demographic variables. Prolific’s platform allows researchers to screen for different demographic information based on previous intake forms that the participants have completed and agreed to be part of future research studies (see also Johnson et al., 2021, for a similar study that used Prolific for participant recruitment). Prolific participants can complete surveys based on a first-come, first-serve basis, if their demographic pre-screen data matches the criteria of a study, and that study is displayed on their personal Studies page. Participants complete the self-administered web-based survey until a study quota has been filled based on participant numbers, and then that study link is closed. In this study, a sample size of 1,500 total (i.e., 500 participants randomized across three study groups) was set as the target goal. A total of 1,555 entries were captured before the study link was closed (i.e., based on the quota). Of these, 44 data entries were incomplete (i.e., blank responses with no results) and discarded, leaving a final total of 1,511 participants. Each participant was paid $1.00 USD (i.e., $10.00 per hour) which was based on Prolific’s standard payment guidelines, and recommended as rates per total survey time, which in this case was ∼6 mins.

**TABLE 1:** Demographic characteristics of participants (*N* = 1,511).

Characteristics	Heterosexual control (*n* = 253)	Heterosexual test (*n* = 250)	Gay control (*n* = 256)	Gay test (*n* = 253)	Genderqueer control (*n* = 256)	Genderqueer test (*n* = 243)
Age (years), mean (SD)	27.1 (8.3)	27.6 (8.4)	28.4 (10.3)	27.3 (9.4)	24.1 (5.7)	24.3 (6.0)
Age range (years)	18–57	18–59	18–65	18–65	18–58	18–54
**Gender**						
Female	185 (73.1%)	187 (74.8%)	160 (62.5%)	159 (62.8%)	0 (0%)	0 (0%)
Male	68 (26.9%)	63 (25.2%)	96 (37.5%)	94 (37.2%)	0 (0%)	0 (0%)
Genderqueer/gender non-conforming	0 (0%)	0 (0%)	0 (0%)	0 (0%)	187 (73.0%)	164 (67.5%)
Trans female	0 (0%)	0 (0%)	0 (0%)	0 (0%)	11 (4.3%)	19 (7.8%)
Trans male	0 (0%)	0 (0%)	0 (0%)	0 (0%)	40 (15.6%)	40 (16.5%)
Different identity	0 (0%)	0 (0%)	0 (0%)	0 (0%)	18 (7.0%)	20 (8.2%)
Gender pronouns*						
She/her	190 (75.1%)	187 (74.8%)	160 (62.5%)	159 (62.8%)	111 (43.4%)	117 (48.1%)
He/him	69 (27.3%)	62 (24.8%)	101 (39.5%)	97 (38.3%)	90 (35.2%)	65 (26.7%)
They/Them	6 (2.4%)	5 (2.0%)	25 (9.8%)	15 (5.9%)	179 (69.9%)	173 (71.2%)
Ze/zir	2 (0.8%)	0 (0%)	2 (0.8%)	1 (0.4%)	9 (3.5%)	3 (1.2%)
Other	0 (0%)	0 (0%)	0 (0%)	0 (0%)	1 (0.4%)	3 (1.2%)
**Sexual orientation**						
Heterosexual	253 (100%)	250 (100%)	0 (0%)	0 (0%)	0 (0%)	0 (0%)
Gay	0 (0%)	0 (0%)	256 (100%)	253 (100%)	50 (19.5%)	47 (19.3%)
Bisexual	0 (0%)	0 (0%)	0 (0%)	0 (0%)	148 (57.8%)	133 (54.7%)
Asexual	0 (0%)	0 (0%)	0 (0%)	0 (0%)	24 (9.4%)	24 (9.9%)
Other	0 (0%)	0 (0%)	0 (0%)	0 (0%)	34 (13.3%)	39 (16.0%)
**Ethnicity**						
White or Caucasian	176 (69.6%)	164 (65.6%)	187 (73.0%)	157 (62.1%)	177 (69.1%)	185 (76.1%)
Hispanic or Latino	29 (11.5%)	30 (12.0%)	20 (7.8%)	31 (12.3%)	24 (9.4%)	16 (6.6%)
Black or African American	16 (6.3%)	21 (8.4%)	18 (7.0%)	23 (9.1%)	13 (5.1%)	11 (4.5%)
Mixed or multi-racial	7 (2.8%)	9 (3.6%)	12 (4.7%)	13 (5.1%)	24 (9.4%)	16 (6.6%)
East Asian	6 (2.4%)	7 (2.8%)	12 (4.7%)	11 (4.3%)	4 (1.6%)	5 (2.1%)
Southeast Asian	6 (2.4%)	6 (2.4%)	4 (1.6%)	13 (5.1%)	10 (3.9%)	3 (1.2%)
South Asian or Indian	9 (3.6%)	6 (2.4%)	1 (0.4%)	1 (0.4%)	0 (0%)	3 (1.2%)
West Asian or Middle Eastern	4 (1.6%)	5 (2.0%)	2 (0.8%)	2 (0.8%)	0 (0%)	2 (0.8%)
Native American or Indigenous	0 (0%)	1 (0.4%)	0 (0%)	1 (0.4%)	4 (1.6%)	2 (0.8%)
Native Hawaiian or other Pacific Islander	0 (0%)	1 (0.4%)	0 (0%)	1 (0.4%)	0 (0%)	0 (0%)

*Note that gender pronouns included multiple selection responses, so numbers may equal over 100% for each study group.

The current study comprised three distinct demographic groups to cover a wide range of different gender identities and sexual orientations. The inclusion criteria for the three study groups were: (i) *Heterosexual–Cisgender* (hereinafter referred to as *Heterosexual*) included a gender identity of male or female, and a heterosexual orientation; (ii) *Gay–Cisgender* (hereinafter referred to as *Gay*) included a gender identity of male or female, and an exclusively gay orientation; and (iii) *Genderqueer* included a non-binary, genderqueer, gender non-conforming, trans male, trans female, or different identity, as well as having a sexual orientation of gay, bisexual, asexual, or other. All pre-screen demographic data as measured by Prolific matched the current study’s question responses with no inconsistencies in self-reported gender identity.

These study groups were used to examine whether minority sexual orientation (*Gay*) or gender identity (*Genderqueer*) alone would independently affect participant attitudes toward pronoun questions compared with a non-minority baseline (*Heterosexual*).

Participants were born in 58 different countries, with most of them from the US (90.6%), and all of them resided in the US, distributed across 52 states and territories. All participants signed informed written consent and the study received full ethics clearance from Canadian SHIELD Ethics Review Board.^2^

### Survey and Procedure

Participants from each demographic group were randomly assigned to complete one of two surveys under the guise of a “personality study for research.” The two different surveys that were developed both included an initial page of demographic questions about age, gender, and ethnicity, but the difference was the inclusion (test version) or exclusion (control version) of the question, “*What are your gender pronouns (select all that apply)?*,” and responses included, “*She/her*,” “*He/him*,” “*They/them*,” “*Ze/zir*,” “*Other.*”

Both study survey versions then included a validated personality test measuring the Big Five dimensions (i.e., Extraversion, Agreeableness, Conscientiousness, Openness, and Neuroticism). This was the Big Five Inventory (BFI) from John et al. (1991) which included 44 items evaluated on 5-point Likert scales from 1 = “*disagree strongly*” to 5 = “*agree strongly*”. Sample items included, “*I see myself as someone who is talkative*”, “*I see myself as someone who is original, comes up with new ideas*”, “*I see myself as someone who perseveres until the task is finished.*” The Big Five Inventory was used to mitigate any suspicion or response bias from the true purpose of the study.

After the personality questionnaire, participants were asked to rate how satisfied they were when responding to the questions in the survey. First, they were asked about their perceptions **specifically on the personality questions** (i.e., Big Five questions). Then, they were explicitly asked another set of questions to rate their perceptions **specifically on the demographic questions** that they answered before. These two separate sets of questions were bolded and underlined as written above so that participants were not confused as to which perceptions they were responding to. Participants were asked to rate each response on a 5-point Likert scale from 1 = “*disagree strongly*” to 5 = “*agree strongly*”, based on the following *ad hoc* statements: “*I felt satisfied answering the questions*”, “*I felt comfortable answering the questions*”, “*The questions were relevant to me*”, “*The questions were important for research*”, “*The questions were easy to understand*”.

Finally, for the control survey version only, participants were asked the same pronoun question as those in the test version, but this was done after survey satisfaction ratings were completed.

Upon completion of the survey, all participants were also given an optional open-ended text box where they could list any other comments or suggestions about taking the survey. This was to examine any qualitative opinions or nuances about the pronoun-related questions, and the survey design in general.

### Data Analysis

**FIGURE 1:**
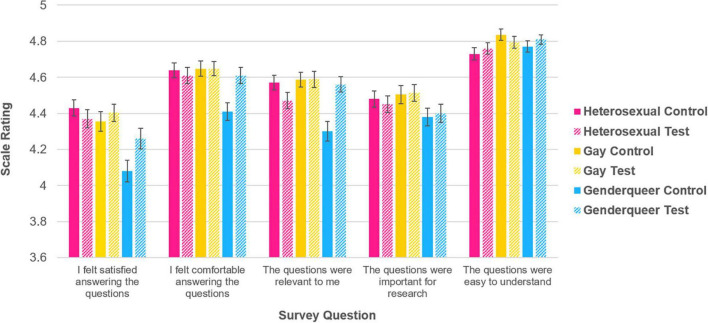
Participants’ attitudes and satisfaction ratings of the demographic questions, as a function of study group and survey condition, shown with standard error bars.

Participants’ satisfaction of the demographic questions was analyzed with a series of 3 (study group: Heterosexual, Gay, Genderqueer) × 2 (survey condition: control, test) between-subjects factorial analysis of variance (ANOVA) using each of the five survey questions (i.e., satisfaction, comfort, relevance, importance, and understanding) as dependent variables (Figure 1).

## Results

### Participant Attitudes and Satisfaction

The main ANOVA revealed significant effects of study group for *satisfaction* [*F*(2, 1505) = 11.337, *p* < 0.0001], *comfort* [*F*(2, 1505) = 5.388, *p* = 0.005], *relevance* [*F*(2, 1505) = 5.980, *p* = 0.003], *importance* [*F*(2, 1505) = 3.137, *p* = 0.044], and a marginally significant effect for *understanding* [*F*(2, 1505) = 2.719, *p* = 0.066]. These results showed that *satisfaction* and *comfort* levels were significantly higher for individuals who identified as Heterosexual and Gay compared to those who identified as Genderqueer (all *p* < 0.05); *relevance* levels were significantly higher for those who identified as Gay compared to Genderqueer (*p* < 0.005); *importance* levels were significantly higher for participants who identified as Gay compared to Genderqueer (*p* < 0.05); lastly, *understanding* levels were marginally higher for those who identified as Gay compared to Heterosexual (all *p* = 0.065).

No significant effects of survey condition were found, but there were significant interactions between study group and survey condition for *comfort* [*F*(2, 1505) = 4.070, *p* = 0.017], and *relevance* [*F*(2, 1505) = 8.790, *p* < 0.0001], and a marginally significant interaction for *satisfaction* [*F*(2, 1505) = 2.647, *p* = 0.069]. Independent samples *t*-tests were performed to assess differences in survey scores between each of the two survey conditions across the three study groups. For participants who identified as Genderqueer, significantly higher scores were found in the test survey condition than in the control survey condition for *satisfaction* [*t*(497), *p* = 0.026), *comfort* (*t*(497), *p* = 0.003], and *relevance* [*t*(497), *p* < 0.001]. No significant differences were found for participants who identified as Heterosexual or Gay.

### Relation Between Age and Perceptions of Demographic Questions

Across the entire sample (*N* = 1,511), age was significantly correlated with importance (*r* = −0.065, *p* = 0.011, and understanding (*r* = −0.088, *p* = 0.001). Age was also analyzed with demographic perceptions for each of the study groups separately. For individuals who identified as Heterosexual, age was significantly correlated with understanding (*r* = −0.089, *p* = 0.046). For individuals who identified as Gay, age was significantly correlated with satisfaction (*r* = −0.105, *p* = 0.017), relevance (*r* = −0.116, *p* = 0.009), importance (*r* = −0.108, *p* = 0.015), and understanding (*r* = −0.109, *p* = 0.014). For individuals who identified as Genderqueer, no significant correlations were found with age.

### Qualitative Opinions

In total, 1,182 participants (78.2%) did not leave a response in the open-ended comment box, while 329 (21.8%) responded with a comment of some kind. Of the 329 who responded, 241 answered “*N/A*” or “*No further comments*”, 38 said “*Thank you*” or “*Good luck*”, and 50 individuals made a comment on the survey or research itself, such as “*I am curious if this relates to Autism Spectrum Disorder based on the personality questions*” or “*reminded me of reading a horoscope*”. Of the 50 individuals who made a comment on the research, six mentioned something specifically about the pronoun question, and all of them were favorable. Participant quotes included:

• *“I like how you included all the pronouns!!!”*
• *“I like that you could select more than one option for pronouns, that was cool.”*
• *“Thank you for having a pronoun choice!”*
• *“I appreciated that I was asked “what are your pronouns” as somebody who is just a little outside of the norm, but not enough to consider myself transgender entirely, I felt able to represent myself accurately.”*
• *“I loved how you could select as many pronouns as applied, that was awesome. Rarely do I get to check all three of mine (he/she/they). Thanks much!”*
• *“Thank you so much for the inclusiveness of the pronoun option! I’ve never seen that in any other survey, and it really made me smile to get to pick like that!”*

Importantly, no participant commented negatively on the use of the pronoun question, and nobody found it offensive or distressing to answer.

### Big Five Personality Dimensions

**FIGURE 2:**
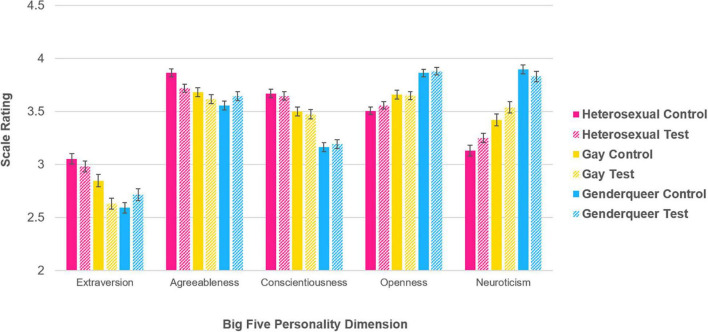
Participants’ big five personality dimension ratings, as a function of study group and survey condition, shown with standard error bars.

Although examining personality differences was not the main purpose of this study, personality scores were also analyzed to provide clarity and interest since these research data were gathered under the premise of “personality study.” Personality dimensions were analyzed with a series of 3 (study group: Heterosexual, Gay, Genderqueer) × 2 (survey condition: control, test) between-subjects factorial analysis of variance (ANOVA) using each of the Big 5 dimensions (i.e., Extraversion, Agreeableness, Conscientiousness, Openness, and Neuroticism) as dependent variables (Figure 2). It is also important to note that demographic factors were not controlled or scaled to any normative database, as is typically done with Big Five dimension analyses (Soto and Jackson, 2013). Results were reported as is due to the inherent demographic differences associated with the three study groups.

The main ANOVA revealed significant effects of study group for *Extraversion* [*F*(2, 1505) = 25.798, *p* < 0.0001], *Agreeableness* [*F*(2, 1505) = 11.876, *p* < 0.0001], *Conscientiousness* [*F*(2, 1505) = 69.115, *p* < 0.0001], *Openness* [*F*(2, 1505) = 43.391, *p* < 0.0001], and *Neuroticism* [*F*(2, 1505) = 91.537, *p* < 0.0001]. Overall, this revealed that *Extraversion* and *Agreeableness* levels were significantly higher for individuals who identified as Heterosexual compared to those who identified as Gay or Genderqueer (all *p* < 0.002); *Conscientiousness* levels were significantly higher for participants who identified as Heterosexual compared to Gay, which in turn, was significantly higher than for those who identified as Genderqueer (all *p* < 0.0001); *Openness* was significantly lower for participants who identified as Heterosexual compared to Gay, which in turn, was significantly lower than for participants who identified as Genderqueer (all *p* < 0.003); lastly, *Neuroticism* was significantly lower for those who identified as Heterosexual compared to Gay, which in turn, was significantly lower than for those who identified as Genderqueer (all *p* < 0.0001).

No significant effects of survey condition were found, but there were significant interactions between study group and survey condition for *Extraversion* [*F*(2, 1505) = 5.161, *p* = 0.006], and *Agreeableness* [*F*(2, 1505) = 4.254, *p* = 0.014]. Independent samples *t*-tests were performed to assess differences in personality dimensions between each of the two survey conditions across the three study groups. For participants who identified as Heterosexual, *Agreeableness* levels were higher for the control survey condition than the test survey condition [*t*(501), *p* = 0.008]. For participants who identified as Gay, *Extraversion* levels were higher for the control survey condition than the test survey condition [*t*(507), *p* = 0.006]. No other significant effects were found.

## Discussion

### Main Findings

This research examined whether the mere presence of a single gender pronoun question within a social research survey increased the satisfaction, comfort level, perceived relevance, and importance of taking that survey within a large sample of heterosexual, cisgender individuals as well as LGBTQIA+ members.

Specifically, for individuals who identified as genderqueer (non-cisgender), significantly higher scores of satisfaction, comfort, and perceived relevance of the questions were elicited when they were given a survey that asked about their gender pronoun versus a survey that did not ask the pronoun question. It is also important to note that no significant differences in Big Five Personality dimensions were found between test and control participants for those who identified as Genderqueer, so it is unlikely that any underlying dispositions of these individuals were the reason for the current findings.

For individuals who identified as heterosexual and gay (cisgender), no major differences were found between survey conditions. This could be due to a ceiling effect since all of the ratings for these groups were quite high, and larger than individuals who identified as Genderqueer in general. It is also possible that being a cisgender individual, regardless of sexual orientation, does not drastically change the perception of typical demographic questions since there is no misalignment between biological sex and social gender identity. This finding supports the results found by Rullo et al. (2018), where no difference in participant attitudes were found between SOGI survey conditions in a predominantly heterosexual and cisgender sample.

Personality differences did exist for participants who identified as Heterosexual and Gay. Participants who identified as Heterosexual had higher agreeableness levels in the control survey condition than in the test survey condition. Participants who identified as Gay had higher extraversion levels in the control survey condition than in the test survey condition. It is unknown why these differences occurred since the Big Five dimensions are considered stable over time, and personality is seen as a trait, rather than a state (Soto and Jackson, 2013). In other words, it is unlikely that personality would be changed from a questionnaire design. Importantly, is that no differences in comfort or satisfaction levels were found between survey conditions for these samples, and it is unclear whether these differences in personality actually influenced any other results since no other differences were found. Future research would have to explore this issue in more detail.

Participants who identified as Genderqueer also experienced the highest levels of neuroticism, which is inline with other research demonstrating personality differences between sexual orientations (Allen and Robson, 2020). Neuroticism is a trait signifying anxiety, self-consciousness, irritability, and emotional instability (Widiger and Oltmanns, 2017). This heightened personality dimension supports the need for pronoun clarification for non-gender conforming individuals who may be more likely to experience questions about their identity and may be denied societal approval.

The results of this study suggest that a non-conforming gender identity specifically, independent from sexual orientation, elicits a preference for pronoun questions and clarification. Additionally, qualitative opinions supported the inclusion of pronoun specification to represent oneself more accurately. This suggests that the collection of pronoun questions in social surveys is received with relatively positive attitudes, and is inline with previous research advocating the collection of SOGI information (Cahill et al., 2014; Westbrook and Saperstein, 2015; Thompson, 2016; Dichter et al., 2018; Haider et al., 2018; Rullo et al., 2018; Grasso et al., 2019; Pinto et al., 2019; Guss et al., 2020; Puckett et al., 2020; Suen et al., 2020; Lau et al., 2021). This experiment highlights the importance of pronoun clarity for genderqueer populations; additionally, for cisgender populations, no negative reaction is likely to be perceived by the inclusion of a pronoun question. In other words, it does not hurt to include a pronoun question in a survey for cisgender individuals, since there is unlikely to be any difference in attitude toward a survey that did not include the same question. Therefore, it may be in surveyors’ interests to include a single, select-all-that-apply, gender pronoun question for all populations.

### Limitations

Some limitations should be noted for this research, including the use of only English-speaking participants from the US. It is possible that other cultures with different languages may be influenced differently by using pronoun questions. For example, certain languages, such as Chinese, have no differentiation between “he” and “she”, whereas other languages, such as French, have many masculine and feminine pronouns to describe everyday nouns. Additionally, western cultures (e.g., North America, Europe, and Australasia) are generally more open to LGBTQIA+ members than non-western societies where sexual and gender minorities can be severely stigmatized. Thus, future research should examine this question more closely across different countries.

Another limitation of this study was the self-administered online implementation of the survey using Prolific’s participant database, which may not generalize to the rest of the larger population. Additionally, 44 entries of the original dataset (*N* = 1,555) were completely blank and discarded by Prolific, so it was not possible to analyze these responses for potential inconsistencies or outliers. However, currently Prolific has approximately 50,000 respondents in the US alone, and the large sample size of this study from 52 states and territories should have helped in recruiting a wide distribution of demographics. Future research would have to replicate this concept with other real-world sample populations, but this study provides insight into a relatively large LGBTQIA+ sample for survey design.

Similar to the limitation of using an online sample, this survey was also self-administered anonymously. Although a main goal was to mitigate any socially desirable responses, it is possible that if participants’ identities were known to the surveyor as in an interview-administrative study, then different results may have been elicited. For example, it is possible that online research may foster fake responses or “troll” participants using an SOGI survey as a platform of mischief (Jaroszewski et al., 2018). Alternatively, SOGI responses may be primed or cause changes in other responses for immediately subsequent items (Cassino and Besen-Cassino, 2021). Importantly, Prolific has several ways of ensuring data quality and validating participants’ true demographics to eliminate any use of bots, cheats, or trolls. Data validation includes having unique participant phone numbers for verification, restricting the number of accounts per IP address, requiring unique PayPal accounts for payment, and continuous examination of survey usage (Bradley, 2018). Additionally, the method of using a “personality survey” in the current study was strategically used to minimize potential priming or downstream effects since there are no “right” or “wrong” answers in the Big Five personality traits, and it is unlikely that someone would want to take a self-report personality questionnaire simply to lie to themselves. That is, participants were recruited based on their pre-registered Prolific profile, which contained their SOGI information, and the study advertisement did not explicitly announce any details about examining gender and sexual orientation. Participants would not have known anything about the true purpose of the study, and instead, they simply completed a survey that was open to them. Lastly, the relatively large sample size of this study should have washed out any inconsistent responses. Overall, the current findings support previous research demonstrating strong support for the inclusion of SOGI questions, especially amongst the LGBTQIA+ population, as these responses clarify any discrepancies between sex assigned at birth, social gender identity, and sexual orientation.

### Conclusion

This research enhances our understanding of the impact of a single pronoun question on participant attitudes of disclosing demographic information, especially for gender minorities. The implications of this research should be used when developing evidence-based policies and procedures for the implementation and collection of SOGI information. This study suggests that the inclusion of a pronoun question may not be distressing or uncomfortable for a wide variety of demographic populations.

The significance of this research is the implication that using gender pronouns during written communication can foster a better culture of DEI principles, and help organizations redesign the way they develop websites, emails, questionnaires, employee applications, and public relations with personnel and clients. Organizational practices should signal identity-safety cues and values supporting LGBTQIA+ individuals with the explicit acknowledgement and inclusion of one’s gender pronouns in organizational materials (Boyland et al., 2018; Gay, Lesbian and Straight Education Network [GLSEN], 2020; Johnson et al., 2021).

All members of society, regardless of heterosexual, cisgender, or LGBTQIA+ status deserve to be recognized, accepted, and feel comfortable during all forms of communication. This research underpins the importance of transparency for gender pronouns, and should be asked whenever possible during daily interactions. It is encouraged that all personal intake surveys and any form of written communication include a personal pronoun question to make members of the community feel welcome and important.

## Data Availability Statement

The raw data supporting the conclusions of this article will be made available by the authors, without undue reservation.

## Ethics Statement

The studies involving human participants were reviewed and approved by Canadian Shield Ethics Review Board. The patients/participants provided their written informed consent to participate in this study.

## Author Contributions

AP programmed the survey, analyzed the data, and contributed to writing the manuscript. LL, AG, and YF helped develop the study idea and contributed to writing the manuscript. All authors contributed to the final review and editing and approved the final manuscript.

## Conflict of Interest

AP, LL, AG, and YF were employed by Klick Health.

## Publisher’s Note

All claims expressed in this article are solely those of the authors and do not necessarily represent those of their affiliated organizations, or those of the publisher, the editors and the reviewers. Any product that may be evaluated in this article, or claim that may be made by its manufacturer, is not guaranteed or endorsed by the publisher.

## Footnotes

1
https://prolific.co/

2
http://cserb.com/
